# Investigating the effective factors and challenges associated with drowning in public pools 

**Published:** 2022-01

**Authors:** Salar Salimi, Parand Pourghane

**Affiliations:** ^*a*^ Student of Pharmacy, School of Pharmacy, Guilan University of Medical Sciences, Rasht, Iran.; ^*b*^ Associate Professor, Social Determinants of Health Research Center, Guilan University of Medical Sciences, Rasht, Iran.; ^*c*^ Associate Professor, Department of Nursing, Zeynab (P.B.U.H) School of Nursing and Midwifery, Guilan University of Medical Sciences, Rasht, Iran.

## Abstract

**Background:**

Drowning in a pool is a rare event that has many negative effects on family members, staff, and the community. The causes and consequences of sinking in the pool are associated with several issues and problems. The purpose of this study is to review the causes of drowning in public pools so that by recognizing and examining the effective causes, appropriate planning can be sought for prevention.

**Methods:**

This study is a review article of published papers (2011-2021) on the impact factors and challenges associated with drowning in public pools. According to the research question, a search was conducted using valid databases of IranMedex, Google Scholar, SID, PubMed, Web of Science, and Scopus to identify studies on the factors and challenges associated with drowning in public pools.

**Results:**

The results showed that the most important factors influencing drowning in public pools are the non-standard space of the pool and lifeguards with insufficient training hours. One of the main problems is the activity of untrained (not trained in lifeguarding) or unqualified (passed the lifeguarding courses with the minimum criteria) lifeguards.

**Conclusions:**

Based on the findings, it can be said that a very high percentage of drowning in swimming pools is preventable and due to the increasing number of swimming pools in the country, the need to check the safety of swimming pools is very important. Also, given the important role of pool lifeguards, they should be adequately trained on the correct methods of rescuing drowning people and performing cardiopulmonary resuscitation. In this regard, careful attention should be paid to holding periodic lifeguard preparation classes and training classes on cardiopulmonary resuscitation scenarios and recording their records, and employing capable people in swimming pools in order to prevent drowning in swimming pools.

**Keywords:**

Drowning, Public Pools, Challenge, Iran

